# Developing and testing an electronic medication administration monitoring device for community dwelling seniors: a feasibility study

**DOI:** 10.1186/s40814-016-0118-3

**Published:** 2017-02-01

**Authors:** Henry Yu-Hin Siu, Dee Mangin, Michelle Howard, David Price, David Chan

**Affiliations:** 1 Stonechurch Family Health Centre, 1475 Upper Ottawa St, Hamilton, ON L8W 3J6 Canada; 2 Department of Family Medicine, McMaster University, Hamilton, Canada; 3 David Braley Health Science Centre, 100 Main Street West, 5th Floor, Hamilton, ON L8P 1H6 Canada

**Keywords:** Medication adherence, Senior adults, Safety, Electronic, Drug monitoring

## Abstract

**Background:**

Medication non-adherence, polypharmacy, and adverse drug events are major healthcare issues leading to significant morbidity, mortality, and healthcare expenditures. Currently, there are no methods to systematically track medication usage in community-dwelling seniors. The eDosette prototype was created to make medication use patterns visible via the Internet. This study aims to demonstrate feasibility, usability, and acceptability of the eDosette in community-dwelling seniors in primary care.

**Methods:**

A 2-week feasibility study involving a convenience sample of 10 patients from an academic family medicine teaching unit in Hamilton, Ontario, Canada, was conducted over a 6-month period between April and October 2015. The eDosette transmitted hourly electronic data via the Internet on each participant’s pattern of medication use; this data was converted into an individualized medication administration record (MAR). Based on the MARs from the 10 participants, the frequency of missed medication doses, the time of dose administration, and each participant’s adherence rate for their prescribed medications could be determined. A medication adherence survey and a patient usability and acceptability survey were administered to all the participants of the study.

**Results:**

The eDosette was able to record a participant’s medication use and transmit this data electronically via the Internet with sufficient quality to create participant-specific MARs. A total of 418 doses were captured by the eDosette throughout the study; only 5% (*n* = 22 doses) were missing information or had poor image quality. Analysis of the MARs revealed that 19% (*n* = 79 doses) were taken outside a 2-h window of the average dose administration time, and two doses were completely missed by all participants during this feasibility study. Participant feedback found the eDosette easy and acceptable to use. Participant feedback also identified hardware and software issues that require attention prior to a larger study.

**Conclusions:**

The eDosette is a feasible and novel technology that can be successfully installed into the homes of community-dwelling seniors to help in monitoring actual medication use patterns. This study provided details for further device development and evidence to support the need for a larger pilot study on the eDosette’s impact on medication adherence.

## Background

Healthcare for senior adults (aged 65 and above) is complex; seniors in developed countries take a median number of seven medications [1, 2]. Medication non-adherence, polypharmacy, and associated morbidity and mortality from drug side effects are major and costly problems for healthcare systems and patients. A Canadian study in 2008 found that 0.75%, or approximately 7000, of annual emergency department (ED) visits in Ontario alone are due to adverse drug events (ADEs) [3]. The estimated cost of these visits in Canadian dollars is $35.7 million per year. Another Canadian ED study reported that 68% of ED visits related to ADEs were preventable [4].

Medication non-adherence may be due to regimen complexity, perceived side effects, lack of information, forgetfulness, cost of medications, and discordance about treatment decisions [5, 6]. Two German studies report that community patients self-report a non-adherence rate of 60% [7, 8]; a Chinese study reported specifically that multi-morbidity is associated with poorer medication adherence [9]. In acute and long-term care settings, medications administered to patients are recorded in a medication administration record (MAR). For community-dwelling seniors, a MAR system does not exist to track how medications are actually taken. As a result, primary care clinicians often do not have objective information on medication adherence during clinical encounters when multiple medication prescriptions are being renewed. Physicians are poor at identifying which patients will be non-adherent to prescribed medications [6]; expecting physicians to accurately and consistently identify non-adherent patients without objective information is not reasonable. Therefore, having information available during medical appointments on how medications are actually used by community-dwelling seniors would greatly assist physicians in identifying those who are non-adherent and could help facilitate treatment decisions and patient engagement in the therapeutic discussion. Reviewing a patient MAR together with the patient may also help clinicians and patients identify individual patient-specific barriers to improving adherence.

The interventions for medication adherence studied in the literature can be classified as either directly or indirectly measuring medication adherence. These interventions range from simple interventions, such as blister packs or dosettes (BP/Ds), to more complex multi-faceted interventions involving behavioural and educational strategies [10–12]. Interventions that directly measure adherence (e.g. direct observation or serum drug levels) are often expensive, impractical, and limited to a single medication [13] while interventions that indirectly measure adherence (e.g. patient questionnaires, pill counting, pill organizers, medication schedules) are plagued by potential bias, false assumptions, and lack of patient engagement and can also be costly [6, 14]. However, there is some evidence that BP/D and other pill organizers increase the percentage of medication pills taken [15].

Incorporating technology is one method to potentially enhance these packaging systems that track medication administration. For example, one device electronically monitors the opening of a pillbox compartment [16], while another intervention provides a custom blister pack enhanced with micro-circuitry to track when a compartment has been punched [17]. These technology-enhanced systems are able to provide objective information on indirect medication adherence; however, their impact on medication adherence has not been adequately studied.

Other technology-mediated interventions involve patient education and self-monitoring to improve medication adherence, with a small proportion focusing on electronic reminder interventions to improve patient adherence [18]. Currently, the main electronic reminder interventions studied are pillboxes with lights and alarms [18]. However, these technology-mediated interventions have not been designed to foster patient engagement in making decisions about their medication regimens. These interventions are also not tethered to a secure online communication method with their primary care team nor with a reporting system for potential side effects. For elderly patients taking multiple chronic medications, interventions with increased physician communication are often necessary to improve adherence [19, 20].

Family physicians are often unaware of medication taking and non-adherent behaviours because of a lack of physician-patient dialogue in community-dwelling seniors [21]. In one primary care study, the least discussed topics in medical encounters include potential side effects, patient attitudes towards current medications, and compliance [22]. The eDosette, the technology-mediated medication adherence intervention presented in this study, was developed to address this primary care knowledge gap regarding the medication use behaviours of older patients. The eDosette is an Internet-enabled device that can monitor medication taking in a patient’s home by sending pictures every hour of the patient’s BP/D to the healthcare provider. The information can then be used to generate the MAR to observe their patient’s medication taking habits. The eDosette is compatible with and can monitor existing BP/Ds, thus eliminating the barrier of requiring patients to use a customized BP/D. The eDosette was also designed with a method for enabling patient engagement and communication through the Internet about their medications, their medication adherence, and potential side effects.

The eDosette device has the potential to be widely used by Canadian seniors despite the fact that it requires an Internet connection. In 2012, the rate of Internet access in Canadian households is 83%, and 59% among seniors. Internet use among seniors in Canada is similar to rates reported in the USA by the PEW Research Centre [23, 24]. Nevertheless, one third of the senior population may not have home Internet; however, Internet access can be made easily available with a cellular hot spot device.

The primary research aims for this feasibility study were to test the eDosette prototype in a primary care setting to determine whether the eDosette could capture and reliably transmit patient medication administration information with sufficient quality over the Internet, to create specific patient MARs detailing actual patterns of medication use, to determine whether the eDosette could transmit potential drug side effect alerts (SEAs) over the Internet, and to identify the technological, hardware, and software issues that may inform future prototype improvements. Additional research aims were to elicit participant feedback about the usability and acceptability of the eDosette and to compare a patient’s actual medication adherence rate to their self-rated medication adherence on the four-item Medication Adherence Questionnaire (MAQ) [25].

## Methods

### Study design

This was a feasibility study of the eDosette in community-dwelling seniors in primary care, conducted between April and October 2015. The intervention lasted 2 weeks, a time frame that was deemed sufficient to determine technological (e.g. data capture and transmission, creation of MARs, Internet connectivity) and user-related issues associated with implementing the eDosette in the home.

### Study population

A convenience sample of 10 patients was recruited from three family practices at an academic family medicine teaching unit in Hamilton, Ontario, Canada. The inclusion criteria were as follows: age 65 and over, taking five or more medications, and using or willing to use a BP/D. Patients with a diagnosis of mild cognitive impairment or dementia were excluded. Potential patient participant lists were generated for the three family practices in the teaching unit; the family physicians identified and approved the patients who were then contacted by telephone for interest in participation by our research assistant (RA). A total of 45 patients were identified and contacted for interest to participate; of these, 35 patients declined participation, resulting in a recruitment rate of 22%. The primary reasons for declining participation during initial telephone contact were lack of interest in participating in general research activities or lack of time. Only two patients declined participation during the first home visit after seeing the device. In one case, their blister pack was too large to fit in the device and the patient declined an alternative compatible device. The second patient declined because they often brought their dosette out of the home (i.e. carried it with them), so their medication administrations could not be accurately monitored. Patient self-perceived competence with using and learning new technology was not an inclusion or exclusion criterion for participation.

### Description of the eDosette prototype

The eDosette device consists of a digital image sensor controlled by a low-cost computer (Raspberry-Pi [26]). The eDosette has a designated compartment where a participant’s BP/D is stored between dose administrations; the image sensor takes serial hourly pictures of the stored BP/D. This continuous longitudinal image data on medication administration is sent via the Internet to the eDosette web-application server where it is accessed and converted into a patient-specific MAR. The eDosette is also equipped with an SEA button, which can be pressed by the patient to indicate to their primary care team about perceived drug side effects in real time. Lastly, a medication disposal unit is included in the prototype; patients can dispose of their unused medications into this unit.

### Study procedures

The first-generation eDosette prototype consists of two versions, a bottom-loading and a top-loading model, to test which design produced higher quality images of the BP/D. Five participants were assigned to the bottom-loading version, and the other five were assigned the top-loading version. Assignment of prototype was based on availability of a top-loading or bottom-loading version upon enrolment in the study. The RA visited each participant’s home to set up the eDosette prototype, provide patient instruction on using the device (e.g. how to load the BP/D into the eDosette, the location, and function of the SEA button, and the eDosette’s medication disposal unit), and assist in finding a suitable location for the eDosette. Overall, the time required for education, set-up, and installation was approximately 20 min per participant. Participants were asked to take their medication according to their usual routine. The RA inspected all the BP/Ds to ensure compatibility with the eDosette; if incompatible, participants were offered a self-fill blister pack or a selection of compatible dosettes for use during this study. Participants managed and stored their medications in the eDosette independently for the length of the trial; this included filling their dosette with the week’s pills or switching out new and old blister packs. All participants were offered the use of a cellular hot spot device to standardize data transmission.

The eDosette was installed in each participant’s home for 2 weeks. The device took an image of the stored BP/D every hour, and the RA used these images to infer when a medication dose was taken. The RA then created a MAR report for each participant, and a second independent coder verified the MARs. The RA performed all data collection. After 2 weeks, the participants were asked to complete a feedback survey, the MAQ [25], and had an exit interview comprised of three questions with the RA about their opinion of the usability and acceptability, as well as their experience with the eDosette prototype.

### Outcome measures

#### Primary outcomes

The primary outcomes consisted of the rate of data capture and transmission over the Internet by the eDosette in the 10 participants, the frequency of SEAs generated, the difference in image quality between the bottom-loading and top-loading first-generation eDosette prototypes, and the identification of technical, hardware, and software issues that arose during installation and use.

#### Secondary outcomes

The secondary outcomes consisted of participant feedback on usability and acceptability, actual participant adherence to prescribed medications determined by preliminary analysis of MARs, and a comparison of participant medication adherence rates as reported by the eDosette MARs to participant self-rated medication adherence on the MAQ [25].

### Data collection and analysis

Each participant’s MAR was reviewed to determine the approximate time of medication administration, the frequency of missed medication doses, and the average variation in timing of each dose administration. Determining the exact time of dose administration was not possible as the eDosette captured images on an hourly basis; therefore, there was no method of determining whether a dose was taken at the beginning or end of the hour (e.g. 09 h05 vs. 09 h55). For this study, medication adherence was defined as the percentage of doses actually administered within a 2-h time window of the average dose administration time; this was based on a previously published definition from electronic adherence monitoring of a single medication that used variation in medication dose timing [27]. Based on this published definition, the investigator group mutually decided that a missed dose or a dose taken 2 h outside the average time of dose administration would be deemed as a significant variation in medication administration.

Participants were asked to complete three items at the end of the study: the four-item MAQ [25], a structured feedback questionnaire, and a very brief interview. The MAQ has been validated in the outpatient setting [28]. The adherence data from the MAR and the participants’ self-rated medication adherence are presented descriptively.

The feedback questionnaire for this study was modeled from an existing survey seeking user feedback after the implementation of a new technology in a healthcare setting [29]. This survey was organized to seek feedback on different domains relevant to evaluating the implementation of a technology in a new setting. The investigator group adapted relevant questions from this survey to use in our eDosette feedback survey, and questions were grouped under six categories: purpose, implementation and usability, impact on daily routine, acceptability for future use, personal opinion, and patient enablement (Table 1). We did not validate the survey in our population. Quantitative survey responses were coded as follows: −2 = strongly disagree, −1 = disagree, 0 = neutral, +1 = agree, and +2 = strongly agree.

**Table 1:** Description of the feedback categories included in the participant feedback survey

Feedback category	Description
Purpose	The investigator group was interested to know whether participants felt the eDosette was able to support participants in managing their medications based on their experience in the 2-week study.
Implementation and usability	The investigator group was interested to know how easy it was for community-based seniors to learn and use the eDosette successfully. In this context, understanding whether the initial meeting and education provided by the RA was sufficient to allow the participant to use the eDosette for the study.
Impact on daily routine	The investigator group was interested to understand the perceived impact of the eDosette on the participants’ lives and roles as patients with respect to medication management.
Acceptability for future use	The investigator group was interested to know whether participants would use the eDosette in the future, outside of the research context. Specifically, understanding whether the technology was a barrier to future acceptability and future use was of interest.
Personal opinion	The investigator group was interested in gaining the personal opinion of participants especially in their perception of whether the eDosette would negatively impact their therapeutic relationship with their healthcare team. Furthermore, the participants were asked to express their satisfaction with the device overall. Additional personal opinions and feedback were elicited during the exit interview with the RA.
Patient enablement	Patient enablement includes confidence and self-efficacy. For the sake of this study, the investigator group was particularly interested in whether the eDosette could result in increased confidence in medication management in our participants.

After the study was completed, the RA asked the participants three interview questions, “What were the three best aspects of the device? The three worst?” and “Do you have any other comments?”. The RA wrote down the responses and clarified the comments with the participant if needed. Participants did not have to supply three answers to each question if they were unable to identify three distinct answers.

Throughout the study, the RA kept a running list of any technical, hardware, or software issues that required discussion and attention among the study team. Minor issues that did not impact the overall design and construction of the prototype were dealt with in real time; for example, longer extension cords were purchased so that possible eDosette locations would not be restricted by proximity to electrical outlets. Issues that would require additional resources or time would be addressed during next-generation prototype development. Both minor and major issues were recorded on an electronic spreadsheet.

Results for medication adherence are presented as mean (minimum-maximum) for continuous variables and as number or percent of participants for categorical variables. For the participant feedback survey, a mean score was calculated for each survey question; a positive mean response indicated agreement with the statement, and a negative mean response indicated disagreement. A formal qualitative analysis was not completed on the participants’ responses from the exit interview; frequency of common statements were collated and reported.

## Results

### Primary outcomes

#### eDosette MAR data

The demographic information for all 10 participants are listed in Table 2. All participants had stable medication regimes during the 2-week study, with no new initiations, titrations, or discontinuations. A total of 418 dose administrations were captured. Information was missing for 22 doses (5%) due to issues with data capture and image quality. Software was initially developed to automatically generate MARs from the transmitted data. However, the software was not able to accurately generate MARs from the data being sent from the participants’ eDosette; likely contributing reasons have been listed in Table 3. As a result, each participant had their 2-week MAR created manually from the transmitted data. To view a sample MAR report, see Table 4. No legitimate SEAs were generated during the 2-week study by any of the 10 participants. One participant initially triggered an SEA each time a medication dose was taken; during follow-up regarding these alerts, it was revealed that the participant had mistakenly understood the function of the SEA button. After this misunderstanding was corrected, no additional inappropriate SEAs were generated.

**Table 2:** Participant demographics

Gender (*n*)	
Male	3
Female	7
Mean age (years, minimum-maximum)	75 (65–87)
Mean number of prescription medications (minimum-maximum)	7.7 (5–10)
Type of medication storage aid (*n*)	
Dosettes (self-filled)	5
Blister packs (pharmacy)	4
Pill bottles (switched to dosette for the study)	1

**Table 3:** Hardware, software, and participant-related issues identified for the eDosette prototype during this feasibility study

Hardware issues	- Could not completely eliminate ambient light from outside the eDosette (major issue) - Could not accommodate all sizes of BP/D currently available on the market (major issue) - The length of the power cord impacted the possible locations of the eDosette in the home (minor issue)
Software issues	Photo-recognition software - Incompatible with some BP/D construction material resulting in poor quality images from the eDosette camera (major issue) - Required BP/D placement in the eDosette to be fixed in a specific location in order for the software to correctly analyse the captured image (major issue) - Could not accurately identify the specific number medications within the BP/D compartment due to the random stacking of medications in the compartment (major issue) Side effect alerts (SEAs) - Some SEAs were sent to the research team by the device without being triggered by the participant (minor issue)
Participant-related issues	- Triggering inappropriate SEA alerts due to misunderstanding the role of the SEA button (minor issue) - Location of BP/D storage for some participants changed as a result of having to locate the eDosette in the participant homes near a wall electrical outlet (minor issue)

**Table 4:** A sample participant medication administration record

		Day of study																
		1	2	3	4	5	6	7	8	9	10	11	12	13	14	15	Mean dose time	VAR (in hours)
Time of dose (24 h time)	Dose 1	10	10	10	7	X	13	16	8	9	11	16	14	9	9	9	11	8.2
	Dose 2	17	15	17	20	X	13	16	16	14	20	16	14	9	16	X	16	8.3
	Dose 3	20	21	20	20	X	22	21	20	20	20	21	X	20	21	X	20 h30	0.45

A participant medication administration record (MAR) showing the time each dose was administered as determined by the images captured by the eDosette. The time listed for each dose administration is in 24 h time (e.g. 20 = 20 h00 = 8:00 pm) and reflects the time of the image when the blister pack or dosette compartment is noted to be empty or partially empty. In this MAR, the “X” indicates missing images. The mean dose time reflects the average time of a particular dose administration over the 2-week study period, rounded to the half hour. Statistical variance (VAR) in the times for each dose was calculated and reported as well

#### eDosette prototype evolution

**Fig. 1:**
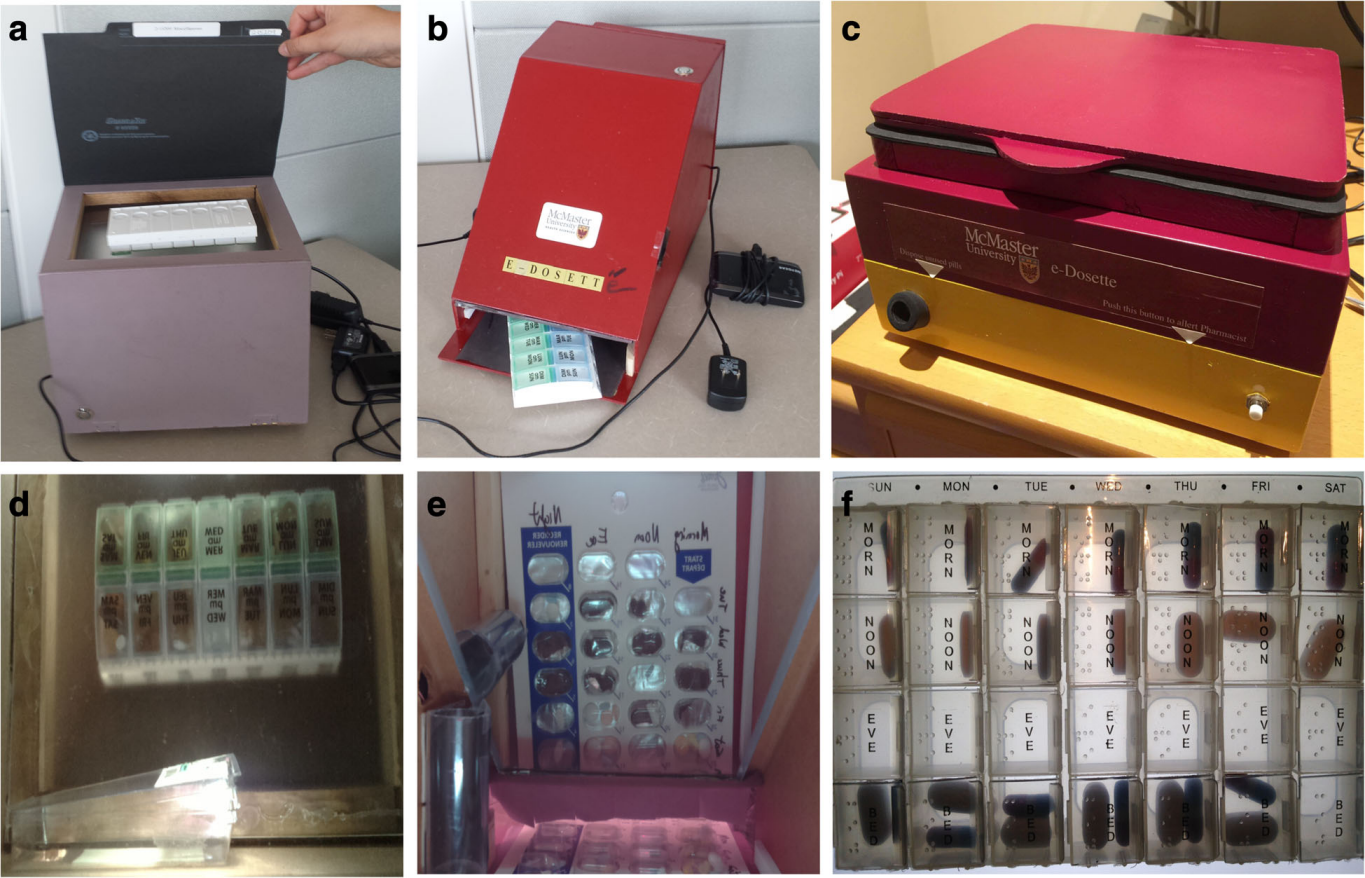
The various iterations of the eDosette prototype and their corresponding image data. **a** The first-generation, top-loading eDosette prototype that includes a side effect alert button and a medication disposal unit. **b** The first-generation, bottom-loading eDosette prototype that includes a side effect alert button and a medication disposal unit. **c** The second-generation, top-loading eDosette prototype that includes a side effect alert button and a medication disposal unit. **d** An example of the image captured by the first-generation top-loading eDosette prototype. **e** An example of the image captured by the first-generation bottom-loading eDosette prototype. **f** An example of the image captured by the second-generation top-loading eDosette prototype

The two first-generation prototypes used in this feasibility study had participants store their BP/D into the eDosette via a bottom-loading (Fig. 1a) or a top-loading slot (Fig. 1b). Half the participants used the bottom-loading model, and the other half used the top-loading model. After analyzing the images captured and transmitted by both prototypes, it was decided that the top-loading first-generation model captured images with better quality. This was likely a result of the top-loading prototype design, which minimizes the distance between the participants’ BP/D and the digital image sensor. The top-loading first-generation model also accommodated a wider range of BP/D types when compared to the bottom-loading model. Furthermore, the bottom-loading slot made it difficult for participants to consistently insert the BP/D adequately into the eDosette to allow for good image capture, and the slot made retrieval of a smaller BP/D difficult. Additional hardware, software, and participant-related issues identified during this feasibility study for both first-generation models are listed in Table 3.

The issues presented in Table 3 led to the development of a top-loading, second-generation model of the eDosette (Fig. 1c). This second-generation prototype was designed and constructed to address issues including blocking out ambient lighting, reconfiguring the storage area to accommodate BP/D of any size or type, and improving the physical aesthetics and size of the eDosette. An example of the image data captured and transmitted is shown under each respective prototype in Fig. 1d–f. The second-generation top-loading model will be the basis for the future planned study on the eDosette prototype.

### Secondary outcomes

#### Medication adherence rates

**Fig. 2:**
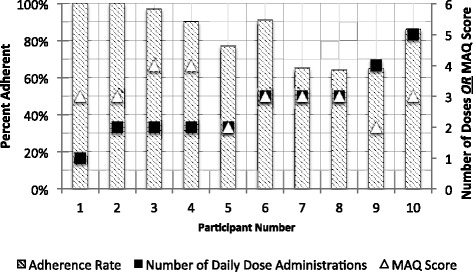
Participant medication adherence during the feasibility study. Comparing medication adherence (i.e. the percentage of administered doses taken within the 2-h time window of the average dose administration time) data to number of daily dose administration for all 10 participants. The Medication Adherence Questionnaire [25] scores for each participant is also shown (0 = low self-rated adherence, 1–2 = medium self-rated adherence, 3–4 = high self-rated adherence)

Overall, two doses were not taken (i.e. missed) by all 10 participants, and a total of 79 doses (19%) were administered outside a 2-h window of the average dose administration time (e.g. a dose taken outside 08 h00–10 h00 for a 09 h00 average dose time). Figure 2 reports observed medication adherence in relation to the number of daily dose administrations and participant MAQ scores. A pattern was observed between the participants’ medication adherence and the number of daily dose administrations; participants with a higher number of daily doses were observed to have lower actual medication adherence as determined by the eDosette. As well, the MAQ score (a higher score indicating higher self-rated medication adherence) was not entirely consistent with the medication adherence reported by the eDosette.

#### Participant feedback survey

On average, participants did not agree with the statement that the eDosette would get them in trouble with their primary care team (mean −1.40) or that having the eDosette in the home felt like someone was watching the participants take their medications (mean −0.70) (Table 5). The mean rating for ease of the use of the technology was 1.20. Mean overall satisfaction with the eDosette was rated 0.50. There was some agreement that the eDosette offered participants a way to be involved in their medications (mean 0.40) and that having an eDosette would positively impact confidence in taking medication correctly (mean 0.60).

**Table 5:** Participant feedback survey responses for the eDosette clustered by feedback domain

Mean score (min, max)	Feedback survey statement	Feedback domain
0.40 (−1, 2)	The eDosette helped me to take my medication more correctly than before.	Purpose
0.30 (−2, 2)	The eDosette made taking my daily medications less confusing.	Purpose
1.20 (0, 2)	I found the eDosette easy to use.	Implementation and usability
1.00 (0, 2)	Training in the use of the eDosette was adequate for me to use it effectively.	Implementation and usability
−1.10 (−2, 0)	I had problems maintaining and taking care of my eDosette.	Implementation and usability
0.40 (−2, 2)	The eDosette allowed me to have a more active role in my medications.	Impact on daily routine
0.20 (−2, 2)	The eDosette made it easier to remember to take my medications.	Impact on daily routine
−0.10 (−2, 2)	The eDosette made my daily medication routine easier.	Impact on daily routine
−0.10 (−2, 2)	With the eDosette, I will rely less on my pharmacy to organize my medications.	Impact on daily routine
0.60 (−2, 2)	I would use the eDosette in the future if it were offered.	Acceptability for future use
−0.60 (−2, 1)	Problems with the eDosette technology would prevent me from using it in the future.	Acceptability for future use
0.50 (−2, 2)	I am satisfied with the eDosette overall.	Personal opinion
0.38 (0, 2)	I feel that with the eDosette, I can now be honest with my family doctor about medication.	Personal opinion
−0.70 (−2, 1)	The eDoesette feels like someone is always watching me when I use medication.	Personal opinion
−1.40 (−2, 0)	I worry the eDosette may get me in trouble with my family doctor.	Personal opinion
0.60 (0, 2)	Did the eDosette increase your confidence in taking medication?	Patient enablement

Responses were coded as follows: strongly disagree = −2, disagree = −1, neutral/did not apply = 0, agree = 1, and strongly agree = 2. Mean scores were then calculated for each question; positive responses indicate an agreement with the statement, while negative responses indicate a disagreement with the statement.

#### Participant feedback from interviews

Participants were asked to list the three worst and best aspects of the eDosette after the trial was completed and to give any other comments about the experiment or device. The RA recorded these responses, and several common comments were identified. The most commonly reported positive aspect, mentioned by half of the participants, was that the eDosette was a consistent place to keep medications and served as a visual reminder for the participant to take their medications. Two participants commented that the eDosette was easy to use. Two participants said that the eDosette helped them realize that they were missing doses, which one said led to better medication taking habits. All participants except one reported positive feedback; the one participant exception only had one daily medication administration. This participant felt that the eDosette did not add to their ability to take their medications.

The participants also identified other negative aspects about the eDosette. Upon seeing the device, all of the participants felt that it was large, and four mentioned this issue in the end interview. Three participants were also discouraged by the inability of bringing the device with them when they were out of their homes. Two participants wanted the device to be able to fit more medications, such as weekly doses, as needed doses or vitamins that were not in their blister pack. One participant was uncomfortable with the idea that medication use was being monitored and felt that the device was very “big brother.”

## Discussion

We conducted a pilot study of the feasibility and acceptability of a device that would send images of BP/Ds of 10 senior adults to the research staff via the Internet over a 2-week period. The recruitment rate was 22%; of those declining participation, most were not interested in research activities in general and the other reasons for declining are outlined previously. This study demonstrated that the eDosette could capture and make visible actual medication use patterns 95% of the time and reported an objective participant medication adherence rate ranging from 64 to 100%. Our observed range is consistent with adherence ranges reported in literature, which are largely based on self-reported adherence measures such as the MAQ [19, 30].

The 2-week trial provided evidence that the technology, even in its rudimentary development stage, was feasible to set up and install in the home of community-dwelling seniors. Setting up the eDosette in participants’ homes required several modifications, such as providing a cellular hot spot device and switching BP/D packaging to be compatible with the first-generation eDosette prototype. The latter issue has been addressed in the second-generation prototype shown in Fig. 1c that has been designed to accommodate the majority of existing BP/Ds. The former issue is a consideration for future prototypes since incorporating a cellular hot spot device into the eDosette could easily be done; however, the cost of this modification and the Internet bandwidth required will need to be assessed in future studies prior to making the addition of cellular hot spot devices a permanent feature on the eDosette. A partnership with telecommunication companies could help reduce this cost and increase the quality of the data transmitted.

Overall, participants only required a short amount of time with the RA, approximately 20 min, to identify a suitable location for the eDosette, become familiar with the device, and successfully use the eDosette for the 2-week study. Participants found the eDosette easy to use and were not worried about the implications of having their primary care team aware of their medication adherence. Interestingly, participants did not strongly endorse statements about the eDosette’s ability to help them take a more active role in their medication use or increase their confidence in using their medication; this was likely due to the fact that the study was focused on testing the eDosette hardware and software. This study was not designed to evaluate whether the eDosette MAR, in conjunction with any resulting clinical communication resulting from reviewing the MAR, would better enable or build confidence in participants while managing their own medications or impact clinical health outcomes.

Even in this small group of participants, the eDosette was able to detect variation in medication taking behaviour that could have implications for effectiveness of the medications. Varying the time of administration within a 2-h window for a medication that is dosed once daily is unlikely to impact the effectiveness of this medication for treating its associated condition. However, this same variation in a multiple daily dosed medication (e.g. the morning dose being taken at 10 h00 vs. 08 h00 and the afternoon dose is taken at 12 h00 vs. 14 h00) may potentially impact the effectiveness of a medication resulting in possible ADEs and participant side effects. Furthermore, the pattern between medication adherence and the number of daily dosages observed in our participants is consistent with findings in the existing literature demonstrating an association between the number of times a day a participant needs to take medications and participant non-adherence to medication regimens [31].

Eight of the participants had an MAQ score of three or higher (Fig. 2), which would indicate good self-rated adherence; however, the range of observed medication adherence in these eight participants ranged from a low of 64% to a high of 100%. This may be a result of the narrow range of possible scores on the four-item MAQ, where each MAQ score may span a wide range of actual observed medication adherence rates. A validated eight-item MAQ exists with a wider range of possible scores; using the eight-item MAQ in a larger study sample may reveal better correlation between a participant’s self-rated and observed medication adherence. Our reported adherence rate is higher than the 47% adherence rate reported in a previous study in senior adults [32]. This study defined adherence dichotomously with a MAQ score of four, as being adherent, and any other score was deemed non-adherent [32]. The MAQ was not designed to be a dichotomous measure [28], and this could contribute to why our reported adherence rate lacked congruence with previously published adherence rates. Furthermore, the previous study did not compare MAQ scores to objective medication adherence data to validate their definition of adherence [32]. A future planned trial of the eDosette, which would include a larger and more representative sample of community dwelling seniors, could potentially report a more accurate estimation of medication adherence rate in these seniors.

### Limitations

The participants in this study were a convenience sample and therefore not randomly selected; as a result, these 10 participants are unlikely to represent the entire spectrum of the senior adult population taking multiple medications. However, as the primary goal of the study was to show that the eDosette could be used in the home to accurately capture and transmit usable image data about medication administration, a truly representative sample of the senior adult population was not necessary at this stage. Our small sample size may not have been large enough to identify all the issues affecting eDosette usability and acceptability; however, the investigator group felt that 10 participants would be sufficient to identify the major issues for the eDosette prototype and inform future prototype development.

Despite the low rate of recruitment, our recruitment strategy was sufficient to recruit the number of participants required for this feasibility study. The reasons given for non-participation may not be reflective of the lack actual participant interest in the eDosette technology but with a lack of time and interest in participating in research in general. As a result, it was not deemed necessary to utilize other methods of recruitment for this study. It is anticipated for the future trial that other strategies will be required; these include collaborating with clinical pharmacists to identify participants, as well as using a generic invitation letter strategy, which is less resource intensive and has been successful in recruitment for other projects led by various members of the investigator group.

The participant exit interview was not a formal qualitative semi-structured interview. The questions posed to the participants may not have allowed for participants to fully express their opinions regarding usability and experience with the eDosette. However, in conjunction with the structured feedback survey, the investigator group felt that the participants were given sufficient opportunity to express their strongest opinions on the eDosette device.

No participants experienced SEAs during the trial; therefore, we could not evaluate the potential of the device to identify side effects experienced by the participants. The lack of reported side effects in this feasibility study could be due to the fact that none of the participants started new medications during their involvement with the study. The apparent lack of clinical decisions resulting from the eDosette MARs in this feasibility study was because the research team reviewed the MARs only to assess the function of the eDosette technology. If the primary care clinicians most involved in the care of the participant had reviewed the MARs instead, it would be possible that clinical issues with medication regimens would have been identified. Clinician involvement and shared decision-making with patients would be one of the key features and outcomes of the future planned trial of the eDosette.

Lastly, the custom photo-recognition software initially designed for the eDosette worked well to generate MARs on standardized laboratory images that were created with controlled lighting and consistent BP/D placement. The software was not able to perform accurately with images captured by the eDosette in participants’ homes. As a result, the research team decided to have two independent reviewers code and manually generate each participant’s MAR for the purposes of this feasibility study. As a result of this adjustment, the research team was not able to truly evaluate how the software performed in automatically generating participant MARs. Although the manually generated MARs for this feasibility study provided accurate medication adherence information, this was at the expense of increased analysis time and would need to be addressed if the eDosette was to be used widely in the community-based setting. With the creation of the second-generation prototype, which addresses the lighting and placement issues identified in the first-generation eDosette, the development of accurate custom software is expected.

## Conclusions

Our study demonstrated that the novel eDosette technology is able to capture and transmit image data of a participant’s BP/D and that the technology can be installed and function in a participant’s home with minimal support. Further work needs to be done to develop software that will automate the MAR generation process, which would significantly improve the clinical usability of the eDosette. This work will be addressed in anticipation of a planned future trial of the eDosette. Lastly, participant feedback and technical issues identified in this feasibility study will lead to further improvements of the eDosette prototype for future trials.

Based on the results of this initial feasibility study, a future trial of the eDosette has been planned, which will include a larger, more diverse sample; will leave the eDosette installed in the home for a longer time period; and will incorporate primary care clinician involvement. Tracking medication adherence and overall medication complexity could be potential outcome measures for this future trial. Participant chart reviews before enrollment in the study and then prospectively throughout the study would allow several other possible outcome measures to be assessed. For example, quantitatively tracking the number of medical appointments or telephone conversations about medication issues specifically could reveal whether the eDosette would impact patient engagement with their primary care clinician. Another example would be tracking the frequency and nature of medication changes during the trial; this could provide information about the types of therapeutic medication decisions, such as dose tapering or dosage time changes, that may be common in the elderly population.

## Abbreviations

ADE: Adverse drug event
BP/D: Blister pack or dosette
ED: Emergency department
MAQ: Medication Adherence Questionnaire
MAR: Medication administration record
SEA: Side effect alert
